# Impact of class-level labelling change on prescriptions of antidepressants for adolescents: An interrupted time-series study using a health insurance claims database in Japan, 2005-2013

**DOI:** 10.1371/journal.pone.0243424

**Published:** 2020-12-07

**Authors:** Yumiko Ogino, Axel Jeremias Schmidt

**Affiliations:** 1 MSc Public Health Programme, University of London International Programme / London School of Hygiene and Tropical Medicine, London, United Kingdom; 2 Department of Public Health, Environments and Society London School of Hygiene and Tropical Medicine, London, United Kingdom; University of South Carolina College of Pharmacy, UNITED STATES

## Abstract

**Background:**

In October 2007, the Japanese Health Authority directed that precautions be added to antidepressants (ADs) labelling regarding suicide risk among young people.

This study evaluates the impact of the labelling change on AD prescriptions and Japanese adolescent suicide rates.

**Methods:**

We compared AD prescription rates per 100,000 population as a primary outcome. The intervention group comprised adolescents (10–24 years), while the control group comprised adults (25–64 years). We defined the pre-intervention period as January 2005 to October 2007 and post-intervention as November 2007 to February 2013. Monthly prescription rate data from a commercial claims database were triangulated with annual suicide rates in Japan. We performed segmented regression analysis for the prescription rates, using a quasi-Poisson model, and tested for level and trend changes.

**Results:**

The commercial claims database included 152,686 adolescents and 195,251 adults during the pre-intervention period and 846,367 adolescents and 1,352,453 adults during post-intervention. Post-intervention, the overall AD prescription rates decreased only in adult males (-95.8 prescription per 100,000) but increased in all other groups. The mean annual suicide rate increased in adolescent males (+1.5 suicides per 100,000) but decreased in all other groups. Overall, the upward trend became moderate or inverse in all groups post-intervention but with a large difference between males and females. The suicide rates rose slightly in adolescents but began declining in adults a year post-intervention. In females, changes in level, trend, and suicide rates were very small in both adolescents and adults.

**Conclusions:**

Contrary to expectations, the mean prescription rates only decreased in adult males, but not in adolescents, regardless of gender. Downward level and trend change were clearly observed in adult males but not in adolescents, the original target of the updated warning. There were no clear temporal associations between suicide rates and the labelling change in either group.

## Introduction

While antidepressants (ADs) can improve depression, several studies have shown that treatment with ADs may increase suicide risk in the young population [1,2]. From 2003 to 2007, the United States Food and Drug Administration (FDA) and the European Medicines Agency (EMA) added strong warnings to AD product information sheets [3,4]. The FDA stipulated that the general warnings about suicide on all AD package inserts should be modified to specify the risk of suicide by paediatric and adolescent patients under 25 years old. This measure was taken in response to an FDA meta-analysis that demonstrated a higher relative risk of suicidality in young people taking ADs compared with those who were given a placebo [5]. In Europe, the precaution regarding suicidal behaviour amongst young people was also added to AD labelling. In response to these regulatory actions, on 31 October 2007, the Japanese Ministry of Health, Labour and Welfare (MHLW) ordered all AD manufacturers to include unified warnings against AD use in young people in the ‘Precautions’ section of package inserts [6]. ADs that entered the market after the intervention also included these warnings in their package inserts.

Several studies in the US, EU, and Australia have already demonstrated that a decrease in rates of AD prescription in adolescents only partially decrease or increase in suicidal behaviour [7–13]. Moreover, spill-over effects on prescription rates were seen in the non-targeted older adult population [7,10,14]. On the other hand, regarding the impact of the same intervention in Japan, one report by the Pharmaceuticals and Medical Devices Agency (PMDA) analysed the Japan Medical Data Center Claims database (JMDC database) [15] and demonstrated that the mean monthly number of paroxetine users aged under 18 years decreased from 13 to 10 users following the labelling change in October 2007. However, it is not known whether these changes in prescription rates resulted in changes to adolescent suicide rates. Therefore, assessing the impact of the intervention is important for successful implementation of future risk minimisation measures.

This study aimed to measure the impact of the labelling change in October 2007 on AD prescription rates and suicides rates among young people aged 10 to 24 years compared with the adult population (25 to 74 years old, control group). The objectives are (1) to analyse the trends of AD prescription rates both before and after the intervention, (2) to evaluate whether the effect varies by gender and drug class, and (3) to examine the potential impacts on suicide rates. The prescription rate was analysed as a process indicator and suicide rate as an outcome indicator for assessing, in accordance with the definition of the EU Guideline on Good Pharmacovigilance Practices (GVP) [16].

## Materials and methods

### Study design

This study comprised a retrospective review of medical records from the JMDC database and Japan’s annual Vital Statistics records by MHLW. The prescription trends were analysed by interrupted time series (ITS). ITS is the strongest design for evaluating longitudinal effects of population-level interventions controlling for prior trends in the outcome [17,18]. To have sufficient power to estimate the parameters, it is essential to have at least 8 time points before and after the intervention in general [19]. When ITS is performed for fewer observations, such as when assessing the effectiveness of interventions immediately after intervention, it requires the analysis of pre-intervention data and stronger counterfactual assumptions or the application of other methods such as sequential monitoring methods [19]. Therefore, the annual suicide rates were not analysed by segmented regression because there were only 3 time points before the intervention and 9 time points in total; however, we compared the mean values of the pre- and post-intervention periods.

### Data sources

Data on AD prescription were obtained from the JMDC database [20], the largest claims database in Japan comprising medical and pharmacy claims data of multiple company employees and their family members from 2005 [21]. The JMDC database does not include data regarding race or ethnicity, but it was assumed that the data are composed mainly of Japanese people as the percentage of foreigners in the total workforce ranged from 0.8% to 1.1% from 2008 to 2013. The database is completely anonymised and standardised. Once retired, members are insured by other insurance schemes; therefore, the JMDC database only includes data of people aged 0–74 years old. Since the JMDC database is based on information from insurance reimbursements, it may not include data regarding all deaths, such as deaths from suicides outside of medical institutions. Therefore, information about suicides was obtained from other sources [21,22]. Annual suicide counts, rates, and data of the population denominator of the whole country were taken from the Japanese Ministry of Health, Labour and Welfare Vital Statistics (Vital statistics) [23].

### Intervention and investigation period

The pre-intervention period comprises 34 months (January 2005 to October 2007). Since another class-level labelling change for precautions for paediatric use was directed by the PMDA in March 2013 [24], the post-intervention period was defined as November 2007 to February 2013, spanning 65 months. Although the PMDA’s pilot study demonstrated a decrease in prescription of paroxetine three months after the labelling change [15], there was no clear time lag between change in labelling and change in prescription rate. Therefore, no time lag was included in the final model. For annual suicide rates, the pre-intervention period was defined from 2005 to 2007, while post-intervention was defined from 2008 to 2013.

### Study population

The target population for the warning is adolescents up to 24 years old. The intervention group was defined as individuals aged 10 to 24 years for the following reasons. Firstly, the WHO definition of adolescents is individuals in the 10–19-year age group [25]. Secondly, ADs are unlikely to be prescribed to infants and very young children. To improve the validity of the results and examine the spill-over effect on non-targeted groups, people aged 25 to 74 years were included as a control group. For analysing prescription rates, the age of patients at time of prescription was calculated on a yearly basis. Each individual was allocated to a certain group based on the time of enrolment in the JMDC database.

### Outcome measures

While the labelling change began with paroxetine (selective serotonin reuptake inhibitor, SSRI) and the group of serotonin-norepinephrine reuptake inhibitors (SNRIs) and was then expanded to all ADs, and because both SSRI and SNRI are recommended as a first-line treatment for mild and moderate depression, we separately investigated two drug categories: ‘SSRIs and SNRIs (SSRI/SNRI)’ and ‘others’ in view of the possibility that the prescribing conditions for patients using SSRI or SNRI and patients using other ADs may differ. AD prescriptions for depression were identified using Anatomical Therapeutic Chemical (ATC) codes (N06: antidepressant). We further stratified AD prescription rates by gender. The drug codes are shown in S1 Table.

As the background trend of depression rates may change over time, we calculated the monthly rate of people with ‘depression’ (F32.x or F33.x) in the JMDC database’s ‘main diseases’ section as depression rates. In addition, some ADs had indications other than depression. Therefore, to avoid selection bias, the number of all enrolees in a month was used as a population denominator.

Suicide rates of the intervention and control groups were calculated from the vital statistics. To remain consistent with the JMDC database, we calculated the rates from the data of adolescents aged 10–24 years as an intervention group and adults aged 25–74 years as a control group.

The number of completed suicides was extracted from the pre-defined category ‘suicides’ as a cause of death, as was the number of individuals in each group. Annual suicide rates were converted per 100,000 people.

### Modelling of segmented regression

A Poisson distribution was used to estimate the monthly number of AD prescriptions. Monthly population denominator data of the JMDC database were included as an offset variable to convert the number into a prescription rate to adjust for an increasing number of enrolees over time.

Bernal et al. provide examples of basic impact models for ITS [18]. The ‘level and trend change’ model was applied based on the hypothesis that the prescription rate increased in line with an increase in the consultation rate for depression. In terms of the trend, the prescription rate rose due to the decreasing stigma of psychiatric disorders, the government’s awareness campaign, and the introduction of new drugs such as SSRIs and SNRIs without a change in labelling (counterfactual) [26,27]. After the labelling change, two hypotheses were made. First, in terms of the actual effect of the labelling change, we hypothesised that the upward trend would be moderate or reversed because physicians would become more careful about administering ADs to young people. In terms of prescription level, the total number of prescriptions (level) would drop as physicians would avoid prescribing to a high-risk population. We hypothesised similar trends as previous studies conducted in other countries [7–13].

In the present study, segmented regression was used to estimate the modelled prescription rates. The following Poisson model (‘level and trend change’ model) was applied [17].

$$Yt=\beta_{0}+\beta_{1}*\text{time}t+\beta_{2}*\text{intervention}t+\beta_{3}*\text{time}\;\text{after}\;\text{intervention}+ET$$

Y*t* represents the log prescription rate at time*t* (log (number of people with ADs / denominator)), where time is a continuous variable to express time elapsed from the start of the observation period; intervention*t* is a dummy variable indicating the pre-intervention period (coded 0) or post-intervention period (coded 1); and ‘time after intervention’ is a counted number of months after the intervention at time *t*. *β*_*0*_ also represents the baseline level of the log prescription rate at time = 0; *β*_*1*_ estimates the underlying pre-intervention (baseline) trend; *β*_*2*_ estimates the level change in log prescription rate following the intervention; and *β*_*3*_ represents the trend change in log prescription rate following the intervention. The trend of post-intervention is calculated as the sum of *β*_*1*_ and *β*_*3*_. *ET* represents the error term.

The relevant data for analysis were extracted from the full dataset of the JMDC database using SAS ver.9.4 and analysed using Stata IC ver.15. *P* values < 0.05 were considered to be statistically significant.

### Sensitivity analysis

Since the depression rate may be a time-variant factor, the number of people with depression was considered to be included in the regression models as an explanatory variable. It is important to check autocorrelations (serial correlations) when conducting ITS because they are assumed to be independent of observations in the standard regression model [17–19]. In the present study, autocorrelation was investigated using correlograms and a Q test for the Ljung-Box test. In addition, seasonal autocorrelation (seasonality) was adjusted by Fourier terms using pairs of sine-cosine [28]. The adjusted models were compared with the unadjusted models using the likelihood ratio test and the Akaike information criterion (AIC) [29].

### Ethical considerations

Ethical approval was obtained from the London School of Hygiene and Tropical Medicine ethics committee (ref.4733/RR/9483). The JMDC database has already been standardised with de-identified individual medical information. As only count data were used, there was no direct patient contact for the data collection and analysis process; therefore, informed consent for the study was not required to be obtained from the patients.

## Results

### Descriptive analysis

During the pre-intervention period, 152,686 individuals aged 10–24 years (adolescents) and 195,251 individuals aged 25–74 years (adults) were included. During the post-intervention period, 846,367 adolescents and 1,352,453 adults were included. The characteristics of the cohorts of each period are shown in Table 1. Background information, such as the proportion of each gender, diagnosis of depression, and the proportion and other frequent psychiatric comorbidities, were similar between pre- and post-intervention periods for both intervention and control groups.

**Table 1:** Baseline characteristics of the cohort.

Adolescents (intervention group)		
	Pre-intervention period N (%)	Post-intervention period N (%)
All	152,686	846,367
Male	86,014 (56.3)	463,273 (54.7)
Female	66,672 (43.7)	383,094 (45.3)
Mean age (SD) at the middle of the period (2006 for pre-intervention, 2010 for post-intervention period)	12.9 (5.11)	16.9 (5.13)
Five most common comorbid psychiatric disorders (ICD-10 Codes)**		
Depression (F32 or F33)	826 (0.54)	5795 (0.68)
Neurotic disorders (F48)	228 (0.15)	2080 (0.25)
Anxiety disorders (F41)	194 (0.13)	2383 (0.28)
Schizophrenia (F20)	153 (0.10)	2061 (0.24)
Somatoform disorders (F45)	141 (0.09)	1475 (0.17)
Adults (control group)		
All	195,251	1,352,453
Male	115,527 (59.2)	755,115 (55.8)
Female	79,724 (40.8)	597,338 (44.2)
Mean age (SD) at the middle of the period (2006 for pre-intervention, 2010 for post-intervention period)	41.7 (10.34)	43.6 (10.7)
Five most common comorbid psychiatric disorders (ICD-10 Codes)**		
Depression (F32 or F33)	6416 (3.29)	45338 (3.35)
Anxiety disorders (F41)	1574 (0.81)	18687 (1.38)
Neurotic disorders (F48)	1222 (0.63)	13309 (0.98)
Somatoform disorders (F45)	992 (0.51)	10527 (0.78)
Schizophrenia (F20)	660 (0.34)	9723 (0.72)

*2006 for pre-intervention period. 2010 for post-intervention period.

**The ICD-10 codes described under ‘main diseases’ of the claims were calculated.

In the intervention group, the mean monthly AD prescription rate increased overall, and for SSRIs and SNRIs, it increased in both males and females, although the increase was more pronounced among males. The mean annual suicide rates increased in male adolescents. Conversely, among adults (control group), the mean prescription rates for AD decreased among men but not among women, whereas the mean suicide rates decreased for both genders (Table 2).

**Table 2:** Changes in mean monthly antidepressant prescription rates.

			Pre-intervention period				Post-intervention period				Change
Adolescents (intervention group)	Prescription rates per 100,000		mean	SD	min	max	mean	SD	min	max	
	Male	Any antidepressants	246.8	21.9	219.1	295.1	295.0	20.3	242.0	338.8	48.2
		SSRI/SNRI	171.2	23.5	127.2	213.4	215.0	17.4	167.8	251.1	43.8
		Other antidepressants	107.0	12.7	84.3	134.1	110.3	11.4	81.0	128.2	3.3
	Female	Any antidepressants	384.4	31.1	326.1	442.1	408.9	22.8	346.3	456.2	24.5
		SSRI/SNRI	289.5	25.1	240.7	338.8	317.7	21.1	266.8	372.0	28.2
		Other antidepressants	148.1	16.6	105.6	188.9	140.1	14.4	108.3	177.3	-7.9
			Pre-intervention period				Post-intervention period				Change
Adults (control group)	Prescription rates per 100,000		mean	SD	min	max	mean	SD	min	max	
	Male	Any antidepressants	1571.1	116.3	1307.9	1719.0	1475.4	91.2	1302.5	1700.2	-95.8
		SSRI/SNRI	1177.1	116.9	941.2	1346.9	1144.1	78.9	994.6	1333.2	-33.0
		Other antidepressants	773.0	35.0	659.8	812.2	643.7	46.0	563.4	787.1	-129.3
	Female	Any antidepressants	1257.0	48.2	1154.1	1335.7	1309.0	36.0	1229.0	1378.9	52.0
		SSRI/SNRI	942.9	55.2	825.3	1024.8	1015.6	27.4	957.2	1060.7	72.6
		Other antidepressants	489.0	21.2	454.2	532.8	489.7	20.6	449.8	529.0	0.7

SD: Standard Deviation; SSRI: selective serotonin reuptake inhibitor; SNRI: serotonin-norepinephrine reuptake inhibitor.

The overall prescription rates were lower than the sum of SSRI/SNRI and other ADs during the investigation period. This result indicated that a substantial number of patients were prescribed both SSRIs or SNRIs, and other ADs (Table 2).

### Modelling of segmented regression

Since the mean was not equal to the variance of the dataset, the scale parameter was added to the model, allowing over-dispersion (quasi-Poisson model). The final models were adjusted for seasonality using the likelihood ratio test, with the AIC showing improvement in the seasonally adjusted models. After adjusting for seasonality, no notable change was observed in the rate ratio and p-values compared with the unadjusted results. The correlograms and Q test showed the presence of an autocorrelation and partial autocorrelation even after adjusting for seasonality. Since the generalised linear model of the Poisson regression used a maximum likelihood estimation, the autocorrelation of data did not affect the results. Although autocorrelation was present in the Poisson regression model, consistent estimates may be obtained using the quasi-likelihood method [30]. The models were not adjusted for depression rates because the correlation coefficient between the number and rates of AD prescriptions and depression indicated strong correlation.

### Overall change in prescription and depression rates

**Fig 1:**
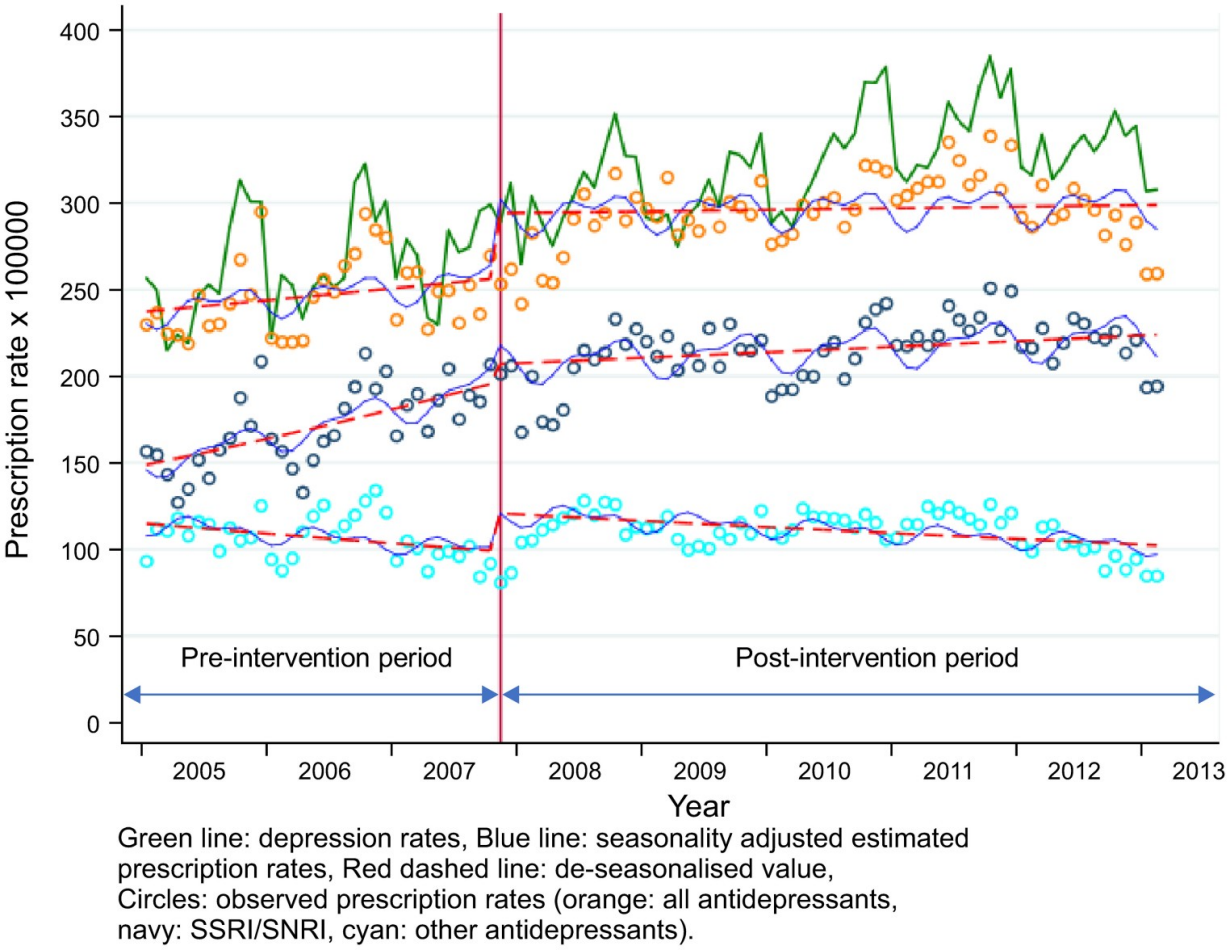
Changes in AD prescription and depression rates among male adolescents (10–24 years, intervention group).

**Fig 2:**
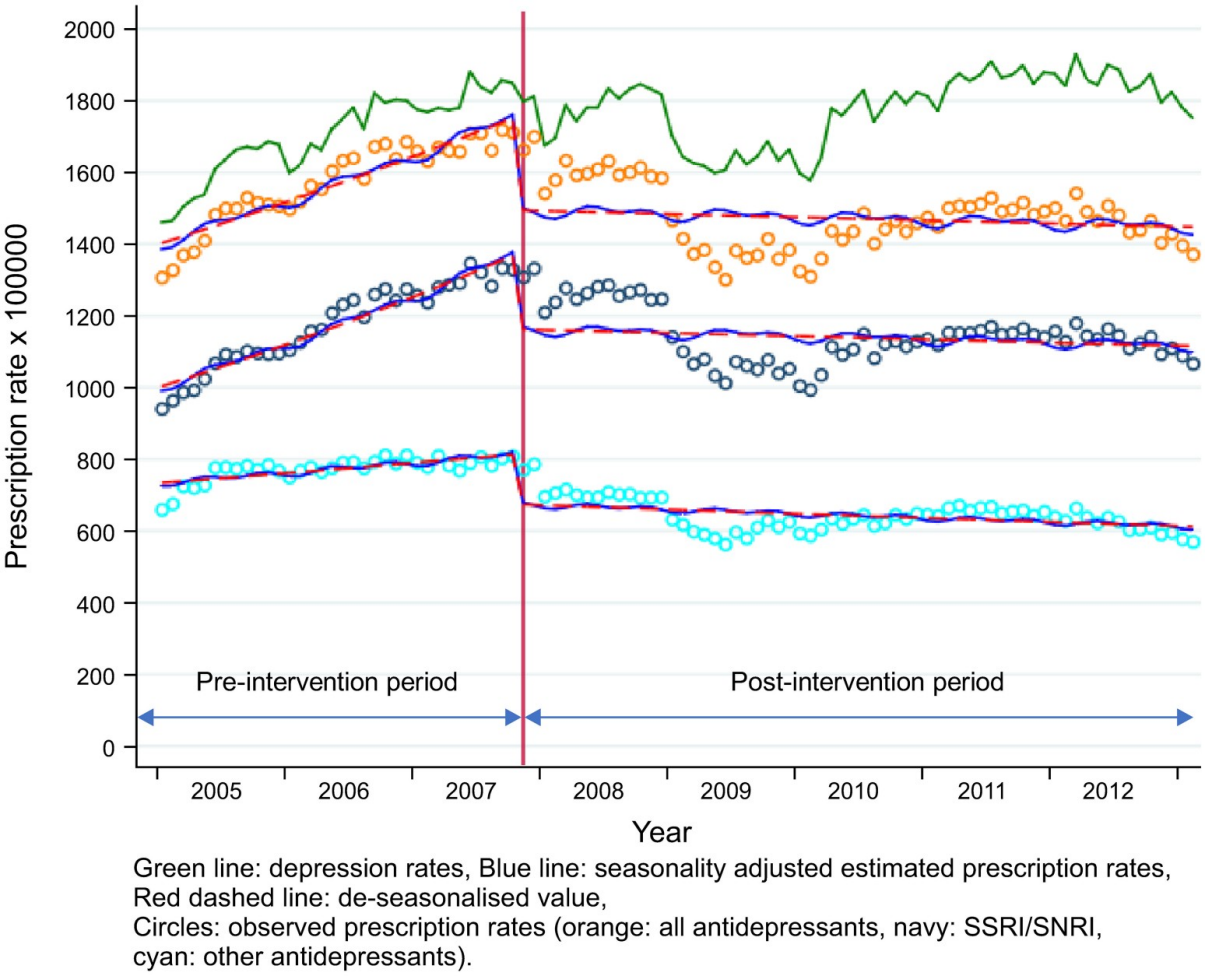
Changes in AD prescription and depression rates among male adults (25–74 years, control group).

When adolescents and adults were compared, a suppressive trend change was observed in both groups after the labelling change. However, the change in prescription level was more prominent in adult males (Figs 1 and 2, and Table 3) and less prominent in females (S1 and S2 Figs and Table 3). The depression rate showed an overall upward trend during the whole investigation period, independent of age and gender in both the intervention and control groups.

**Table 3:** Segmented analysis of antidepressant prescription rates after the labelling change.

	Males				Females			
	RR	*P-*value	95%CI		RR	*P-*value	95%CI	
Adolescents (10–24 years, intervention group)								
Any antidepressants								
Baseline level *β*_0_*	237.1	<0.001	217.9	258.0	352.1	<0.001	331.9	373.5
Baseline trend *β*_*1*_	1.002	0.255	0.998	1.006	1.005	<0.001	1.002	1.008
Change in level *β*_*2*_	1.148	0.001	1.060	1.242	1.021	0.456	0.966	1.079
Change in trend *β*_*3*_	0.998	0.321	0.994	1.002	0.994	<0.001	0.991	0.997
SSRI/SNRI								
Baseline level *β*_0_*	147.7	<0.001	134.8	161.8	265.0	<0.001	247.6	283.7
Baseline trend *β*_*1*_	1.008	<0.001	1.004	1.013	1.005	0.002	1.002	1.008
Change in level *β*_*2*_	1.058	0.173	0.975	1.148	1.038	0.255	0.974	1.106
Change in trend *β*_*3*_	0.993	0.001	0.989	0.997	0.994	0.001	0.991	0.998
Other antidepressants								
Baseline level *β*_0_*	115.5	<0.001	102.9	129.5	146.9	<0.001	133.2	162.0
Baseline trend *β*_*1*_	0.996	0.115	0.990	1.001	1.000	0.837	0.996	1.005
Change in level *β*_*2*_	1.220	0.001	1.087	1.368	1.051	0.314	0.954	1.156
Change in trend *β*_*3*_	1.002	0.529	0.996	1.007	0.996	0.108	0.991	1.001
Adults (25–74, control group)								
Any antidepressants								
Baseline level *β*_0_*	1393.5	<0.001	1321.1	1469.9	1213.8	<0.001	1172.3	1256.7
Baseline trend *β*_*1*_	1.007	<0.001	1.004	1.009	1.002	0.021	1.000	1.004
Change in level *β*_*2*_	0.854	<0.001	0.810	0.900	1.009	0.623	0.975	1.044
Change in trend *β*_*3*_	0.993	<0.001	0.990	0.995	0.998	0.033	0.996	1.000
SSRI/SNRI								
Baseline level *β*_0_*	994.4	<0.001	938.7	0.0	866.2	<0.001	836.4	897.1
Baseline trend *β*_*1*_	1.009	<0.001	1.007	1.012	1.005	<0.001	1.003	1.006
Change in level *β*_*2*_	0.850	<0.001	0.804	0.899	0.995	0.772	0.962	1.029
Change in trend *β*_*3*_	0.990	<0.001	0.987	0.993	0.995	<0.001	0.994	0.997
(continued)								
Baseline level *β*_0_*	732.4	<0.001	693.7	773.2	513.0	<0.001	488.4	538.9
Baseline trend *β*_*1*_	1.003	0.021	1.000	1.006	0.997	0.023	0.995	1.000
Change in level *β*_*2*_	0.830	<0.001	0.785	0.878	1.073	0.006	1.020	1.129
Change in trend *β*_*3*_	0.995	0.001	0.993	0.998	1.002	0.062	1.000	1.005

RR: Rate Ratio 95% CI: 95% confidence interval. All models were adjusted for seasonality.

*Converted to per 100,000 individuals.

### Change in prescription rates

#### Adolescents (10–24, intervention group)

The results of the final model of the intervention group are shown in Fig 1 and Table 3. Overall, the pre-intervention (baseline) trend in prescription rate for any ADs increased by an average of 0.36% per month. After the intervention, the level of AD prescriptions increased by 8.2%, and there was a downward change in the trend (0.4% per month) after the intervention. Therefore, the total prescription rate decreased by 0.04% per month after the labelling change.

A difference was observed in terms of gender and drug classes. In female adolescents (Table 3 and S1 Fig), an upward trend was inverted after the labelling change. The level increased but not to a statistically significant level. The same trend was also found in SSRIs/SNRIs, but not in other ADs.

In male adolescents (Fig 2 and Table 3), a large level change (14.8% increase) was observed in all ADs. A larger level change was found in other ADs (22.0% increase) with no significant trend change, whereas the change in level did not significantly increase in SSRI/SNRI prescriptions. However, the trend of SSRI/SNRI prescriptions changed from a sharp increase in the pre-intervention period to moderate in the post-intervention period.

The gap between depression rates and prescription rates of all ADs widened after the labelling change, particularly in the intervention group; this indicated that the number of adolescents who were depressed but were not administered ADs increased after the intervention.

#### Adults (25–74, control group)

The results of the final model of the intervention group are shown in Fig 2 and Table 3. However, the scatter plot of the prescription rates reported in the control group demonstrated a slightly non-linear pattern during the investigation period.

A considerable difference was observed between males and females. A significant decrease in level was noted in males, due to the prescription of both SSRIs/SNRIs and other ADs (Fig 2 and Table 3). Fig 2 also shows an immediate decrease in prescription rates after the labelling change in 2007. Furthermore, in 2009, an additional decrease in prescription rates was observed in males. By contrast, no significant level change was observed in the prescription rates of ADs in adult females (Table 3 and S2 Fig).

### Change in suicide rates

**Fig 3:**
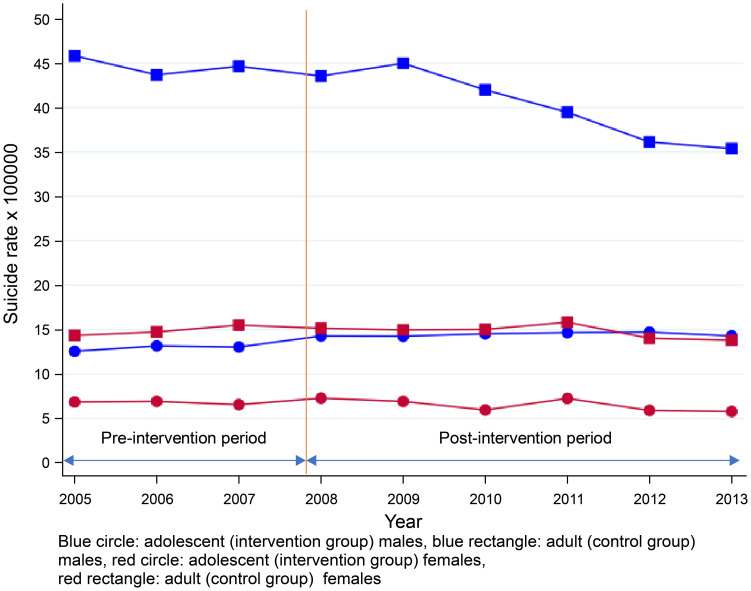
Changes in suicide rates.

During the study period, the suicide rate was remarkably higher in adult males than in other groups. From 2005 to 2009, the suicide rates were stable in all groups, followed by a decrease in the rate of adult male suicide but not amongst other groups (Table 4 and Fig 3). After the stratification by age group (10–14, 15–19, and 20–24 years), no group demonstrated a clear downward trend. A slight increase was noted among males aged 20–24 years (S2 Table).

**Table 4:** Changes in mean annual suicide rates.

		Pre-intervention period				Post-intervention period				Change
Adolescents (intervention group)	Suicide rates per 100,000	mean	SD	min	max	mean	SD	min	max	
	Male	12.9	0.3	12.6	13.2	14.5	0.2	14.3	14.7	1.5
	Female	6.8	0.2	6.6	6.9	6.5	0.7	5.8	7.3	-0.3
Adults (control group)	Male	44.7	1.1	43.7	45.8	40.2	3.9	35.4	45.0	-4.5
	Female	14.9	0.6	14.3	15.5	14.8	0.8	13.8	15.8	-0.1

## Discussion

### Principal findings

Our findings demonstrated a prominent decrease in prescription rates amongst adult males, who were not the main target of the added warnings. The prescription rate of all drug classes decreased immediately after the intervention in adult males, whereas the level increased slightly in adolescent males. In relation to trend change, in both the intervention and control groups, the sharp upward trend in AD prescription, particularly in the SSRI/SNRI group, became a decrease in incline in the upward trend or reversed to a downward trend after the labelling change. However, the extent of trend change was similar. In addition, suicide rates in young populations were steady after the labelling change, and such rates increased slightly in 2009.

The impact on prescription and suicide rates observed in this investigation exhibited both similarities and differences to those reported in previous studies in other countries. Similar additional warnings on labelling suppressed the increasing trend in prescription rates in targeted and non-targeted populations in some studies [7,8,10,14]. In contrast, in Canada, SSRI prescription increased in the young population from 2005 to 2009, after the addition of the FDA warning. Of note, the approval of the paediatric indication of several SSRIs influenced their increased use in Canada [31]. In Japan, although the efficacy and safety of SSRIs for paediatric patients has not been established, the use of psychotropic medications, including ADs, has considerably increased. That is, the number of patients aged <20 years seeking medical treatment for psychiatric disorders increased from 95,000 in 2002 to 148,000 in 2008 [32]. A retrospective study has shown that SSRIs were widely used not only for mood disorders but also used off-label for other psychiatric disorders in children aged 7–18 years [33]. In addition, 26% of paediatric patients who used antipsychotics also used them in combination with ADs [32]. In contrast to the ‘black box warning’ at the top of labelling in US packaging, the wording of the precaution added to Japanese package inserts does not prohibit the use of ADs in young individuals in Japan. Therefore, the interpretation of the precaution may vary between physicians and countries depending on underlying trends of the incidence of depression and suicide and on the languages in the labelling.

The present study demonstrated a slight decrease in the suicide rates of young populations in 2009, which is when the global economic crisis occurred. However, the level of AD use decreased in the US, and the increase in the rates of suicide attempts occurred simultaneously with the decreased use of ADs in the young population after the addition of the warning by the FDA [7,14]. In the present study, on the other hand, even the prescription rate rose after the labelling change. It is unclear whether the increased suicide rate was a result of the increasing rate of untreated depression. Although no decisive factor can explain why the level change was more prominent in males in the control group, there were some potential factors related to the obtained results. First, the main objective of the labelling change was to warn about the increasing risk of suicide among adolescents. In addition, the revised wording for suicide risk in the important precautions section is not specific to the young population but can be applicable to all populations. Therefore, physicians are reminded of the risk not only to adolescents but also to adults, and they also prescribed ADs to adult males more cautiously because this group has the highest suicide rate in Japan. Second, another labelling change in 2009 warned against the harmful behaviour and clinical worsening (including suicidal behaviour) related to AD use in all populations [34,35], and this may have contributed to the decrease in the prescription rate, particularly in the control male group. The effect of this labelling change would lead to an overestimation of the decreased modelled rates of prescription during the post-intervention period. Third, the observed prescription rates decreased again from 2012 to 2013. The introduction of guidelines for the treatment of depression in 2012 advised not only of the careful administration of antidepressants to patients with mild depression, but also regarding the assessment of the risk of suicidality due to AD use [36]. These events may have amplified the negative trend during the post-intervention period, particularly in adult males.

The causal relationship between depression and suicide has been well established; however, other factors such as financial issues, unemployment, challenges in personal relationships, and problems related to school have also been linked to suicidal behaviour [37,38]. The impact of the labelling change on suicide rates may be limited due to the complexity of causal pathways and interaction of suicide risk factors. Of note, the global financial crisis affected Japan’s economy in 2008, leading to increased unemployment in the young population (S3 Table). Consequently, job insecurity and economic worsening may have maintained the high rates of suicide observed in the young population. In contrast, the decreasing rates of suicide among adult males since 2009 were analysed, and the results demonstrated that interventions that provide social supports and economic recovery contribute to improvements [39].

### Strengths and limitations

The present study has several strengths. Firstly, it is appropriate for evaluating the effectiveness of the labelling change as both process and outcome indicators are discussed [16]. Secondly, the study design is ITS, which is the strongest quasi-experimental design to assess the impact of an intervention in non-randomised settings [17]. Finally, the main study outcomes were to ascertain the actual numbers and rates of prescription, which are less prone to reporting and observer bias.

However, this study also has some limitations. Firstly, unemployment is a known risk factor for depression and suicide [40]. However, the JMDC database did not contain data regarding self-employed and unemployed individuals, who are at a relatively higher risk of depression and suicide than corporate workers due to financial insecurity and psychosocial stress. Secondly, suicidal behaviour such as suicidal thoughts and self-harm have not been investigated as surrogate outcomes due to the difficulty of obtaining such data from the claims database. Thirdly, the enrolees in the JMDC database may differ slightly from the overall population in Japan. The household income, extent of job insecurity and poverty, level of education, and living conditions of enrolees in the JMDC database may differ from individuals covered by other social health insurance schemes. Fourthly, the JMDC database contained potential measurement biases such as coding inaccuracies and underreporting, which claims databases have by their nature [14]. Therefore, the diagnosis of depression could be more than the actual number given, because patients who did not need medication would not include the codes for depression if it was not required for reimbursement. Furthermore, the use of the segmented regression method has limitations, such as being affected by rapidly changing, time-varying confounders, and long-term non-linear patterns [17,18]. In addition, reductions in prescription at the individual level (i.e. reduction of dosage, number of concomitant ADs, and interruptions) were not investigated. Furthermore, severity of depression is not included in the JMDC database; therefore, background changes in the overall severity of depression or differences in severity among drugs were not investigated. Regarding suicide rates, the impact of other policies to prevent suicide cannot be excluded. This includes part of a national health promotion policy named ‘Healthy Japan 21’ that allocated budget for suicide prevention in 2001, a new law called ‘General Principles of Suicide Prevention Policy’ in 2007, and a funding programme for local governments.

Further studies are required to validate the reasons for the large shift observed in control group males. To investigate the influencing factors, information on the specialities of prescribers can clarify whether drugs were prescribed by psychiatrists or other health workers and whether they were prescribed before or after a diagnosis of depression. Qualitative studies targeting physicians could also help to understand why they changed or did not change their prescriptions. The gap between depression rates and prescription rates of all ADs widened after the labelling change, particularly in the intervention group; however, it is not clear whether patients who were not prescribed ADs remained untreated or received alternative therapies. The long-term impact of AD prescription in young individuals on population health and suicide rate should be assessed in future studies. To eliminate the uncertainty caused by using different databases, it would also be helpful to use alternative endpoints of suicides that can be captured in the claims database such as emergency treatments for overdoses.

## Conclusions

Contrary to expectations, a negative level change in the prescription rate was clearly observed in adults, particularly in males, compared with adolescents. A similar negative trend change was observed in both groups. A possible explanation was that physicians have been reminded about the association between medication and suicide risk by the revised precautions, and as a result, prescribed ADs to adults more cautiously because this group has the highest suicide rate in Japan. In contrast, there seems to have been no clear temporal association with suicide rates.

## Supporting information

S1 Fig Changes in prescription rates and depression rates among female adolescents (10–24 years, intervention group). (TIF)

S2 Fig Changes in prescription rates and depression rates among female adults (25–74 years, control group). (TIF)

S1 Table Drug codes and categories. (DOCX)

S2 Table Annual suicide rates per 100,000 adolescents, by age group. (DOCX)

S3 Table Annual unemployment rates per 100,000 by age group. (DOCX)
