# Supplementary figures and images for: Impact of class-level labelling change on prescriptions of antidepressants for adolescents: An interrupted time-series study using a health insurance claims database in Japan, 2005-2013
Source: PLoS One. 2020 Dec 7;15(12):e0243424. doi: 10.1371/journal.pone.0243424 (PMC7721198; doi:10.1371/journal.pone.0243424)

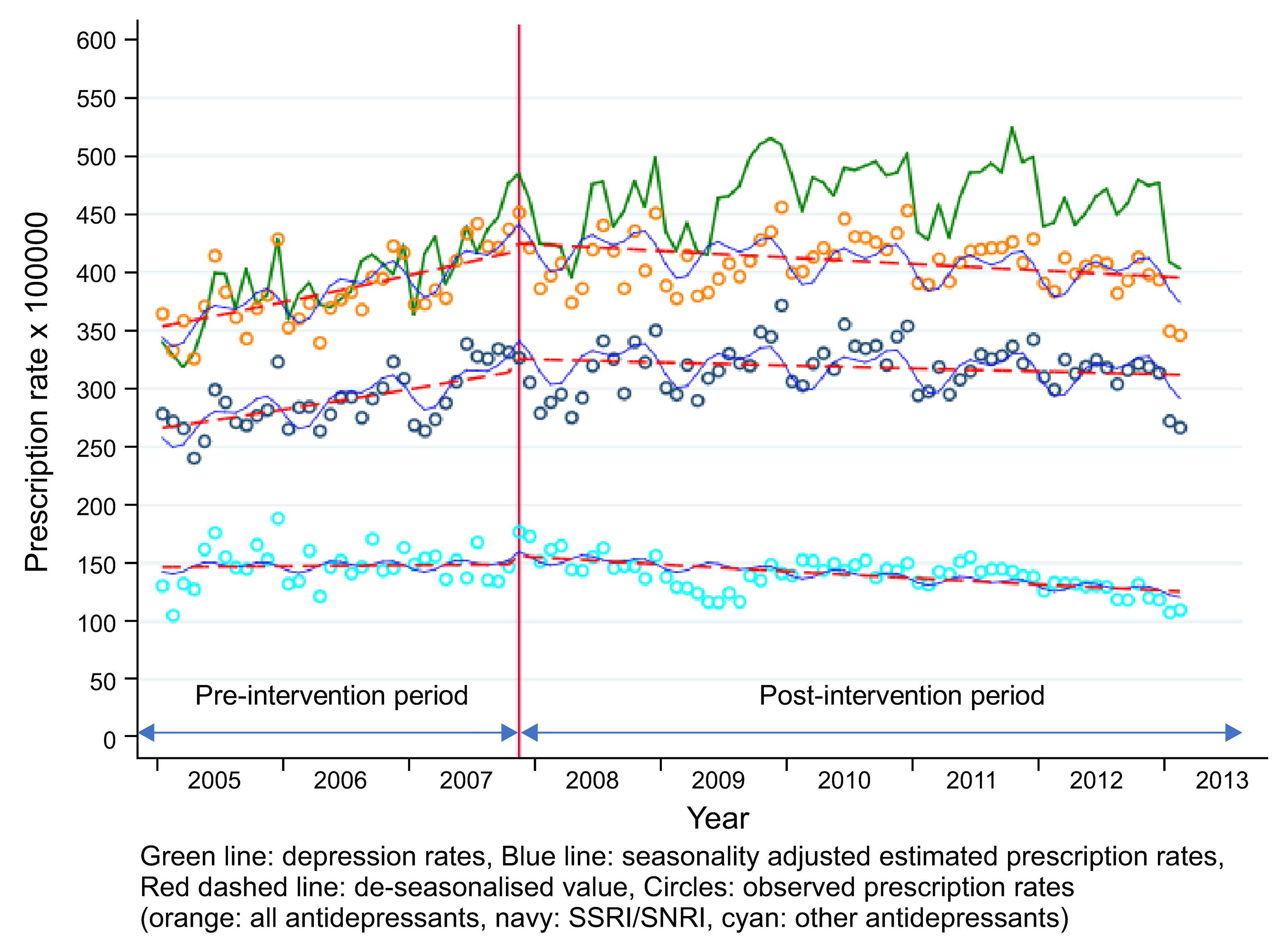

Supplement: S1 Fig — (TIF) [file pone.0243424.s001.tif]

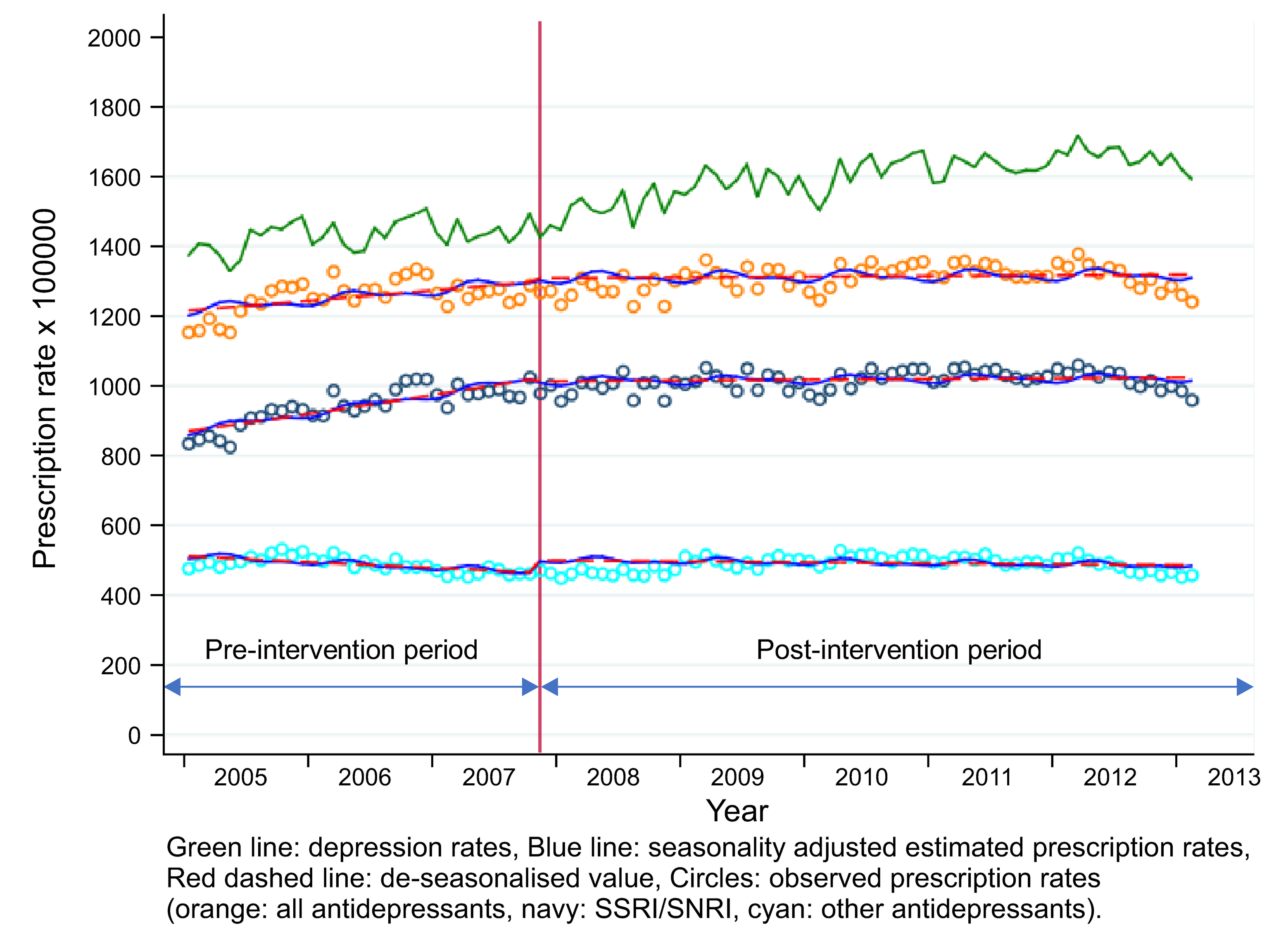

Supplement: S2 Fig — (TIF) [file pone.0243424.s002.tif]
