# Supplementary material for: Impact of class-level labelling change on prescriptions of antidepressants for adolescents: An interrupted time-series study using a health insurance claims database in Japan, 2005-2013
Source: PLoS One. 2020 Dec 7;15(12):e0243424. doi: 10.1371/journal.pone.0243424 (PMC7721198; doi:10.1371/journal.pone.0243424)
Supplement: S1 Table — (DOCX) [file pone.0243424.s003.DOCX]

## S1 Table Drug codes and categories

| Generic name | ATC code | drug category | drug class |
| --- | --- | --- | --- |
| sertraline | N06AB06 | SSRI | SSRI/SNRI |
| fluvoxamine | N06AB08 | SSRI | SSRI/SNRI |
| paroxetine | N06AB05 | SSRI | SSRI/SNRI |
| escitalopram | N06AB10 | SSRI | SSRI/SNRI |
| milnacipran | N06AX17 | SNRI | SSRI/SNRI |
| venlafaxine | N06AX16 | SNRI | SSRI/SNRI |
| duloxetine | N06AX21 | SNRI | SSRI/SNRI |
| clomipramine | N06AA04 | TCA | Others |
| amitriptyline | N06AA09 | TCA | Others |
| amoxapine | N06AA17 | TCA | Others |
| lofepramine | N06AA07 | TCA | Others |
| imipramine | N06AA02 | TCA | Others |
| trimipramine | N06AA06 | TCA | Others |
| nortriptyline | N06AA10 | TCA | Others |
| dosulepin | N06AA16 | TCA | Others |
| mirtazapine | N06AX11 | TeCA/NaSSA | Others |
| maprotiline | N06AA21 | TeCA | Others |
| setiptiline | - | TeCA | Others |
| mianserin | N06AX03 | TeCA | Others |
| trazodone | N06AX05 | partial SSRI* | Others |

*Trazodone is not categorised SSRI in the Japanese package insert despite the mechanism.

SSRI: Selective Serotonin Reuptake Inhibitor, SNRI: Serotonin Noradrenalin Reuptake inhibitor

TCA: tricyclic antidepressant, TeCA: Tetracyclic antidepressant, NaSSA: Noradrenergic and Specific Serotonergic Antidepressant
