# Supplementary material for: Impact of class-level labelling change on prescriptions of antidepressants for adolescents: An interrupted time-series study using a health insurance claims database in Japan, 2005-2013
Source: PLoS One. 2020 Dec 7;15(12):e0243424. doi: 10.1371/journal.pone.0243424 (PMC7721198; doi:10.1371/journal.pone.0243424)
Supplement: S2 Table — (DOCX) [file pone.0243424.s004.docx]

## S2 Table Annual suicide rates per 100,000 adolescents, by age group

|  | Males | | | Females | | |
| --- | --- | --- | --- | --- | --- | --- |
| Year/Age | 10-14 | 15-19 | 20-24 | 10-14 | 15-19 | 20-24 |
| 2005 | 0.91 | 9.57 | 24.99 | 0.55 | 6.00 | 12.90 |
| 2006 | 1.83 | 9.94 | 25.52 | 0.69 | 5.70 | 13.27 |
| 2007 | 1.02 | 8.93 | 26.79 | 0.55 | 5.66 | 12.46 |
| 2008 | 1.35 | 9.83 | 29.33 | 0.59 | 6.79 | 13.53 |
| 2009 | 1.12 | 9.66 | 29.93 | 0.73 | 5.47 | 13.74 |
| 2010 | 1.39 | 9.72 | 31.41 | 0.73 | 5.12 | 11.64 |
| 2011 | 1.73 | 11.08 | 30.44 | 0.77 | 5.71 | 14.93 |
| 2012 | 1.84 | 11.28 | 30.41 | 0.70 | 5.63 | 11.18 |
| 2013 | 1.94 | 10.48 | 29.89 | 1.21 | 4.60 | 11.36 |

Source: the Vital statistics
