# Supplementary material for: Impact of class-level labelling change on prescriptions of antidepressants for adolescents: An interrupted time-series study using a health insurance claims database in Japan, 2005-2013
Source: PLoS One. 2020 Dec 7;15(12):e0243424. doi: 10.1371/journal.pone.0243424 (PMC7721198; doi:10.1371/journal.pone.0243424)
Supplement: S3 Table — (DOCX) [file pone.0243424.s005.docx]

## S3 Table Annual unemployment rates per 100,000 by age group

| Year/Age | 15- 19 | | 20-24 | | 25-29 | Whole population |
| --- | --- | --- | --- | --- | --- | --- |
| 2005 | 10.2 | 8.4 | | 6.2 | | 4.4 |
| 2006 | 9.4 | 7.7 | | 6.0 | | 4.1 |
| 2007 | 8.7 | 7.5 | | 5.7 | | 3.9 |
| 2008 | 8.0 | 7.1 | | 6.0 | | 4.0 |
| 2009 | 9.6 | 9.0 | | 7.1 | | 5.1 |
| 2010 | 9.8 | 9.1 | | 7.1 | | 5.1 |
| 2011 | 9.2 | 8.2 | | 6.5 | | 4.6 |
| 2012 | 7.9 | 7.9 | | 6.4 | | 4.3 |
| 2013 | 6.4 | 7 | | 6.2 | | 4.0 |

Source: Labour Force Survey in 2014 (Ministry of Internal Affairs and Communications).
